# Guiding the emerging primary care researcher: A report of research capacity-building workshop

**DOI:** 10.4102/safp.v65i1.5769

**Published:** 2023-12-20

**Authors:** Shane D. Murphy, Arun Nair, Ramprakash Kaswa, Indiran Govender, Klaus von Pressentin

**Affiliations:** 1 Department of Clinical Services, Abbey House Medical Centre, Navan, Ireland; 2 Department of Family Medicine, University of the Free State, Kimberley, South Africa; 3 Department of Family Medicine and Rural Health, Walter Sisulu University, Mthatha, South Africa; 4 Department Family Medicine and Primary Health Care, Sefako Makgatho Health Sciences University, Pretoria, South Africa; 5 Division of Family Medicine, University of Cape Town, Cape Town, South Africa

**Keywords:** research, capacity-building, clinician-scientist, education, family medicine

## Abstract

There is an escalating interest in research capacity building across the globe. Research is an integral part of the continuous improvement process, clinical decision making and health system strengthening and should be embedded into the health system. The *South African Family Practice Journal* editorial team held a workshop on 19 August 2022 at the 24th National Family Practitioners Congress in Cape Town, with the aim of supporting primary care clinicians in their development from early-career researchers to established clinician-scientists. Small group and plenary discussions yielded valuable insights into the lived experiences of early career researchers and highlighted critical action areas to build the landscape of clinician-led research in the South African context.

**Contribution:**

This article contributes to current literature by providing insight into the lived experiences of early-career researchers and explores opportunities for research capacity-building.

## Background

Primary health care professionals need to implement new, context-specific evidence to improve the health of their patients and communities. Research grounded in clinical care should be embedded into the health system, with clinicians actively involved in research to drive continuous improvement processes and health systems strengthening.^1^ Furthermore, contextualised research ensures that the evidence base used to inform practice and education remains relevant. In addition to improving healthcare outcomes, clinician-scientists in primary care support both self-directed and team learning while developing competencies such as critical thinking, change agency, increased uptake of evidence-based practice and higher levels of job satisfaction.^1^ The benefits of clinical research further cascade to the health system in the form of service plans and policymaking, informed by a bottom-up evidence base.

The escalating interest in enhancing research capacity building (RCB) for clinicians on an international platform has focused on infrastructure, skills training and best practices.^1,2^ However, context-specific RCB in developing countries requires a broader approach to facilitate sustainable capacity building that remains contemporaneous and ethnographically relevant.^3^ Research capacity building is described as a:

[*D*]ynamic intervention operationalised through a range of foci and levels to augment the ability to carry out research or achieve objectives in the field of research over the long-term, with aspects of social change as an ultimate outcome.^4^ (p. 2),^5^ (p. 2)

In the South African context, RCB has not enjoyed sustained investment in its development and implementation among local roleplayers. This is compounded by the fact that there are limited opportunities for early career researchers to meet and learn from each other and more experienced researchers. However, it is a critical gap to address as inequalities in health research contribute to inequalities in health.^6^

The South African Family Practice (SAFP) editorial team aims to support colleagues in their development from early-career researchers to established clinician-scientists. This report aims to capture the key lessons learned during an in-conference workshop at a national conference in family medicine and primary care.

## Workshop process

A 2-h research capacity-building workshop for early-career researchers was held on Friday, 19 August 2022 at the 24th National Family Practitioners Congress in Lagoon Beach Hotel, Cape Town.

The workshop aimed to stimulate interest in research and develop capacity among early-career clinician-scientists, by identifying opportunities, resources and techniques to commence their research journey as part of their engaged scholarship portfolio. The workshop was attended by five facilitators from the SAFP editorial team and 14 attendees, together representing nine institutions and three Southern African countries (South Africa, Lesotho and Namibia). The attendees included six undergraduate (UG) students from two universities in the Western Cape, one registrar in family medicine, one Doctor of Philosophy (PhD) student and six postgraduate (PG) students.

The workshop began with a brief introduction of attendees and sharing experiences. This was followed by a 40-min breakaway into two groups. Group members were randomly allocated, which allowed a mix of backgrounds and experiences in each group (see Table 1). The groups were asked to:

- *Describe common difficulties encountered when starting off in the research world*.
- *Explore ways to make time for research as a busy clinician*.
- *How to explore formal and informal development opportunities for early career researchers*.
- *How to identify and engage research partners, research networks and mentors*.

**TABLE 1:** Activities planned for the workshop.

Time in minutes	Activities
10	Introduction and overview of the need for primary care scholarship.
10	Short reflections on experiences of what helped them start a journey in primary care research, shared by two early career researchers.
40	Group work (in a small group format): each small group focuses on one of the workshop objectives.
30	Small group feedback – plenary.
15	Reflection on the group work insights (buzz pairs).
15	Planning of next steps and linking with the community of practice. Sharing of take-home messages.

Following the group work, all attendees reconvened for plenary feedback and finished with take-home steps and linking and/or networking for a community of practice. Table 1 highlights these activities.

The comments and suggestions from both groups’ discussions, as well as the plenary session, were documented on flip charts (during group activities) and collated during the plenary session. The audio recording of the session was reviewed by the facilitators alongside the collated data to provide thick descriptions of the attendees’ discussions under each objective.

## Workshop outputs

The following outputs are described according to the objectives of the workshop.

### Common difficulties encountered when starting off in the research world

A major challenge described by attendees was the unfamiliarity of setting out on a research journey: ‘… we don’t know what we don’t know’ (UG1).

Attendees described uncertainties, as well as a lack of confidence, with the early building blocks of research: choosing a research topic, how to develop a research question and what research methodology to use. Several attendants cited insufficient exposure as UG students and currently viewed the process of research as ‘… the whole nightmare of logistical challenges, ethics approval … and data collection’ (PG1).

Several questions arose around the concepts of research methodology and considerations common to new researchers: ‘…we need supervision, particularly related to the research question’; ‘… is a simple QIP research, or do you have to go much deeper than that … much bigger?’ (UG4).

In addition, attendees described a sense of academic isolation, reporting an unawareness of academic structures – who to contact and how. A sense of frustration became apparent as attendees described their efforts to reach out to senior researchers were not responded to: ‘… there is a lack of connectivity between the higher-ups and those who are more junior’ (PG2).

On a personal level, attendees were hesitant to either start research, or continue with a project if any challenges arose – ‘we have expectations of a Utopian experience of research … we want it to be perfect, but it’s maybe messier than we think it is’ (PG4). This thought was shared by several attendees. They proceeded to outline that research was a learning curve and that disappointment was a part of the learning process – ‘You need to manage your expectations while you research …’ (PG3) and that feedback, although hard to hear, was formative – ‘… remember to receive criticism openly, don’t become disheartened … Become familiar with your own strengths and weaknesses’ (PG5).

### Making time for research as a busy clinician

Attendees commonly cited a lack of dedicated time for research activities. Attendees felt that the workplace demands for clinical services span the time and energy available. However, the discussions quickly became solution-driven: ‘It’s not all doom … there are many things we can do to create time for research …’ (PG4).

Respondents felt that they could better utilise their time with prioritisation of research-dedicated time when creating a work schedule. Outside of their own capacity to find time for research, attendees felt that ‘systemic changes that introduced time dedicated to research even for those not at academic settings … protected time agreements from line managers’ (UG2) was necessary as well.

Another suggestion made by attendees was that of researching topics of personal interest – ‘… research something that you have a passion for … you will automatically find time’ (PG1).

This suggestion was coupled with a pragmatic solution: aligning research projects with workplace activities – ‘… kill two birds with one stone … align your research to your daily duties’ (PG5).

Some attendees felt that they were able to be agents of change, to create a culture of research among colleagues within their workplace and peer groups – … we talk about healthy competition … surround yourself with people who are also doing research’ (UG4). This sentiment was echoed by others who held that we needed to be proactive when building our capacity as researchers: ‘When you have an opportunity to join a research project that is not necessarily your own, join it … you get to see the research unfolding and learn how to do it yourself’ (PG3).

### Exploring formal and informal development opportunities for early career researchers

Attendees viewed both formal and informal development opportunities as critical to personal research building capacity. However, they described a realisation that formal opportunities were easier to conceptualise and discuss: ‘formal research retreats … workshops … academic days where you present progress reports or actual academic work’ – (PG3). There was strong support for the integration of research development opportunities into both registrar-linked training programmes as well as independent workplaces: ‘Systemic changes to encourage academic leaders/influencers to drive awareness around research … create positions dedicated to helping early-career researchers’ (UG1).

Various suggestions were put forward by the group; however, they lacked description or clear actionability: ‘… they should develop structures that promote research and entry into a world of research’ (UG6) *and* ‘Expand the pool of mentors and supervisors, as well as provide links for communication between mentors and prospective researchers’ (UG4).

When probed on these suggestions, there was an apparent lack of awareness around academic support structures and organisations. However, informal development opportunities arose as realisable actions/goals: ‘… people could maybe do a part of the research’ (UG3)*and* ‘… peer-reviewing as a way to build up your ability to do research’ (PG2). These steps were seen as continuous learning opportunities to build research capacity: ‘… building your toolbox of research skills … you must learn from all of the failures on the way …’ (PG1).

Supervisors and mentors were viewed as critical to the learning process: ‘… see one, do one … and also getting feedback on what you’re doing’ (UG4) and ‘we need motivation and support … formal [*supervisor*], informal – use whatever friend you have’ (PG2). However, attendees also described a sense of responsibility towards establishing development opportunities within their environments: ‘Find like-minded people in your workplace … such as journal club to stimulate academic work … encourage each other, share learnings and challenges’ (UG1).

### Identifying research partners, research networks and mentors

In comparison to the previous topics where attendees easily voiced their opinions, the start of this discussion point was met with hesitation and uncertainty: ‘… not knowing where to start … who to speak to …’ (UG6) and ‘There are people who want to do research, … and there are people who want to mentor and teach … but often there’s a disconnect as those people don’t meet’ (PG2).

Gradually, the uncertainties were met with pragmatic solutions to address the unmet needs of early clinician researchers: ‘… clarify your research topic and seek out a mentor with similar interests’ (PG2) and ‘… identify researchers with a “good track record” based on the number and quality of publications … area of interest’ (PG4). Notably, attendees spoke to the development of ‘… authentic, value-based relationships with mentors’ (UG3) to form long-term synergistic partnerships where mentors were ‘… willing, and keen to provide guidance’ (PG6).

The SAFP workshop on research-building capacity was described as an example of a useful networking opportunity: ‘Workshops like today, with the contact details on the attendance research ‘… we can reach out … that’s how we can make our own network …’ (UG1) and called for more frequent ventures to build research capacity: ‘… roadshows by academic partners to create awareness, drive shared-learning and explore research networks and partnerships’ (PG3).

## Discussion

The workshop groups that participated came from diverse backgrounds (three countries) and represented various levels of academic pursuits across the continuum of learning (from UG to PhD levels). This allowed for an array of different experiences and reflections to be shared.

Insufficient exposure to research at the UG level was a prominent hindrance shared by this group of early-career researchers. This created uncertainty around how to get started with research in their settings. This finding is similar to local^7^ as well as international^5,8^ studies where an absence of exposure to research during UG studies resulted in a reluctance to commence research by clinician-scientists in their settings. Where research is integrated into UG curricula, clinicians describe a sense of self-awareness of personal research competencies (i.e., strengths and weaknesses) and the resultant insight into research methodology instilled a sense of empowerment to conduct research in clinical practice.^9^ This may be attributable to enhanced confidence in personal capabilities and reduced apprehension around research methodologies, data management, ethical aspects and whom to contact or get guidance from to proceed in a path previously less travelled.

Another major obstacle to building research capacity was the time needed to pursue a research journey, especially competing workplace demands, such as clinical service delivery and other family or social responsibilities. The culture of research was felt to be better supported in academic training institutions than the common general practice or primary care settings that were more service delivery orientated. This obstacle is common across the globe, but more pronounced in low- and middle-income countries (LMICs) where human resources are overburdened.^6,10^ While effective time management (at the level of the individual) was highlighted by some attendees, the call for a system-level policy that allows dedicated time in the workplace was held as fundamental to enabling research activities,^8^ including those who need to perform research as part of their formal studies. Academic and governmental policy support allowing dedicated time has been shown to be a strong enabler of enhancing research capacity in developing countries.

Consistent with studies in LMICs where clinician RCB was underdeveloped, our attendees also described a lack of awareness around research support networks.^8,11^ Few were aware of how to get into special interest groups or join existing research platforms or networks to seek guidance or inspiration to pursue research interests. This sentiment highlights the systemic under-appreciation of clinician-scientist research that has led to a limited pool of mentors or supervisors who have the time to commit to prospective early career researchers. Not knowing whom to contact or not getting a response when reaching out to potential mentors, is demotivating as clinicians embark on their research journeys.^12^ This challenge is compounded by the paucity of health research leadership in the Global South,^13^ which is meant to open up opportunities for research funding and development.

The SAFP workshop was described as a useful networking opportunity where early career researchers could meet and connect with others with similar backgrounds and the editor team of the SAFP. Getting to meet the editor team of a journal they wish to publish in also removed anonymity and improved trust and confidence in approaching the journal. There was a general feeling that such workshops and even road shows will assist early career researchers to build confidence and negotiate common difficulties encountered when starting off in the research world. While insufficient as a stand-alone activity, workshops are fundamental to RCB initiatives in the South African context because of their ability to increase peer networking, sharelearning opportunities and create a professional dialogue among early career clinician-scientists.^7^ Workshops such as these may also contribute to the creation of networks and communities of practice, especially via an online platform, which has been shown to be feasible and acceptable in the African region.

### Recommendations

Multi-level initiatives are critical to embedding research into clinical practice. Academic, governmental and private partnerships are necessary to drive sustained investment in RCB. Top-down initiatives such as expert consensus statements to inform policy reform that supports research should be coupled with workshops such as the SAFP workshop where the lived experiences of early career researchers are expressed.

Research capacity building must be prioritised across UG academic programmes as well as national health priority setting. Continued investment in embedding research into UG programmes is critical to creating a culture of continuous improvement in the health sector. Student-led initiatives,^14^ journals and societies at institutional, national and international levels^14,15,16^ have also shown benefits in growing scholarly interest and capacity.

The governmental departments of higher education and health, professional associations and academic institutions, as well as scientific journals, should actively increase the pool of research mentors and leaders through active recruitment, peer review skill workshops and mentorship training. Furthermore, fora for generating interprofessional dialogue and networking will enhance awareness of the availability of resources to support early career researchers. Professional associations in family medicine and primary care such as the SAAFP should link with regional and international bodies aimed at advancing the scholarly foundation of academic primary care, such as the World Family Doctors Association (WONCA), working party on research,^17^ the Primafamed network,^18^ as well as similar groups such as the Society of Academic Primary Care (SAPC)^19^ and North American Primary Care Research Group (NAPCRG).^20^

## Conclusion

The workshop held by the SAFP editorial team at the 24th SAAFP Conference enjoyed a varied group of attendees who shared useful insights into the lived experiences of early career clinician-scientists. The workshop highlighted key areas to address to support the research initiatives of clinician-scientists in their everyday environment. Sustained investment in RCB and its respective support structures at the UG and graduate levels is needed to match the growing interest in primary care research among early-career researchers.
